# Cost-effectiveness and budget effect of pre-exposure prophylaxis for HIV-1 prevention in Germany from 2018 to 2058

**DOI:** 10.2807/1560-7917.ES.2019.24.7.1800398

**Published:** 2019-02-14

**Authors:** David A M C van de Vijver, Ann-Kathrin Richter, Charles A B Boucher, Barbara Gunsenheimer-Bartmeyer, Christian Kollan, Brooke E Nichols, Christoph D Spinner, Jürgen Wasem, Knud Schewe, Anja Neumann

**Affiliations:** 1 Viroscience department, Erasmus Medical Centre, Rotterdam, The Netherlands; 2 Institute for Health Care Management and Research, University of Duisburg-Essen, Essen, Germany; 3 Department for Infectious Disease epidemiology, Robert Koch Institute, Berlin, Germany; 4 Department of Global Health, Boston University, Boston, United States; 5 Health Economics and Epidemiology Research Office, Department of Internal Medicine, School of Clinical Medicine, Faculty of Health Sciences, University of the Witwatersrand, Johannesburg, South Africa; 6 Department of Medicine II, University Hospital Klinikum rechts der Isar, Munich, Germany; 7 dagnä (Deutsche Arbeitsgemeinschaft niedergelassener Ärzte in der Versorgung HIV-Infizierter), Berlin, Germany

**Keywords:** HIV, men-who-have-sex-with-men, MSM, preexposure prophylaxis, PrEP, cost-effectiveness, prevention, Germany, sexually transmitted infections, viral infections, acquired immonodeficiency syndrome, AIDS, HIV infection, infection control, public health policy, modelling

## Abstract

**Background:**

Pre-exposure prophylaxis (PrEP) is a highly effective HIV prevention strategy for men-who-have-sex-with-men (MSM). The high cost of PrEP has until recently been a primary barrier to its use. In 2017, generic PrEP became available, reducing the costs by 90%.

**Aim:**

Our objective was to assess cost-effectiveness and costs of introducing PrEP in Germany.

**Methods:**

We calibrated a deterministic mathematical model to the human immunodeficiency virus (HIV) epidemic among MSM in Germany. PrEP was targeted to 30% of high-risk MSM. It was assumed that PrEP reduces the risk of HIV infection by 85%. Costs were calculated from a healthcare payer perspective using a 40-year time horizon starting in 2018.

**Results:**

PrEP can avert 21,000 infections (interquartile range (IQR): 16,000–27,000) in the short run (after 2 years scale-up and 10 years full implementation). HIV care is predicted to cost EUR 36.2 billion (IQR: 32.4–40.4 billion) over the coming 40 years. PrEP can increase costs by at most EUR 150 million within the first decade after introduction. Ten years after introduction, PrEP can become cost-saving, accumulating to savings of HIV-related costs of EUR 5.1 billion (IQR: 3.5–6.9 billion) after 40 years. In a sensitivity analysis, PrEP remained cost-saving even at a 70% price reduction of antiretroviral drug treatment and a lower effectiveness of PrEP.

**Conclusion:**

Introduction of PrEP in Germany is predicted to result in substantial health benefits because of reductions in HIV infections. Short-term financial investments in providing PrEP will result in substantial cost-savings in the long term.

## Introduction

Sex between men is the predominant route of transmission of human immunodeficiency virus (HIV) in Europe [1]. In Germany, the number of new HIV diagnoses among men who have sex with men (MSM) remains high, with almost 2,000 new diagnoses in 2016 [2], despite the availability of condoms, high coverage of antiretroviral drug treatment for individuals diagnosed with HIV and high levels of virological suppression in people using treatment. The large number of new diagnoses indicates that additional HIV prevention approaches are needed. One such prevention method, pre-exposure prophylaxis (PrEP) with the antiretroviral drugs tenofovir disoproxil fumarate (TDF) and emtricitabine, has been shown to prevent new HIV infections in MSM at high risk of infection [3-5]. In trials, PrEP reduced the risk of HIV infection by 85% irrespective of whether it was used daily [4] or on-demand, where individuals took a double dose 2–24 h before sexual contact and two single doses 24 and 48 h later [5]. Community studies have also shown high PrEP effectiveness among individuals vulnerable to HIV infection in real-world settings [6].

The high cost of PrEP was until 2017 a primary limitation for its use in HIV prevention [7]. The patent on TDF and emtricitabine, however, expired in 2017, resulting in a generic price of EUR 50 per 28 days in Germany, which was more than 90% lower than the EUR 700 per 28 days that had to be paid for branded TDF and emtricitabine [8]. Despite this strong reduction in costs, PrEP is not reimbursed in Germany and eligible individuals have to pay for PrEP out of their own pocket. Use of PrEP can, however, be associated with substantial societal health benefits by averting HIV infections [4,5]. In addition, preventing new infections by introducing PrEP could result in financial societal benefits as individuals living with HIV require life-long treatment which contrary to PrEP is fully reimbursed in Germany.

The aim of this study was to assess the cost-effectiveness and budgetary effect of introducing PrEP in Germany. For this purpose, we used a mathematical transmission model [9]. The model includes the individual HIV-preventive benefit of PrEP among MSM at high risk of HIV infection, and the population preventive benefit of PrEP that is due to reducing the number of secondary infections in populations where PrEP is introduced [9].

## Methods

### Mathematical transmission model 

For this study, we adapted an existing mathematical transmission model from the Netherlands [9] to the HIV epidemic among MSM in Germany. The Dutch and the German HIV epidemics are comparable with nearly 70% of infected individuals in both countries reporting MSM as the mode of transmission [2,10]. The German HIV epidemic is well described by the national Robert Koch institute, which collects epidemiological information including key parameters such as the number of new diagnoses and the route of transmission [11].

### Model assumptions and calibration

The Dutch mathematical model was adapted using parameters that represent the German HIV epidemic among MSM from 2013 to 2015 (Table and Supplement). The model stratifies disease progression into the acute stage, three chronic stages (CD4^+^ T-cell count > 500 cells/μL, CD4^+^ T-cell count 350–500 cells/μL, and CD4^+^ T-cell count 200–349 cells/μL) and one acquired immunodeficiency syndrome (AIDS) stage (CD4^+^ T-cell count < 200 cells/μL). The schematic representation of the model and the equations can be found in the Supplement pp 1–6. Each stage of infection has a different duration and infectivity (Table) [12,13]. We assumed that antiretroviral drug treatment for HIV reduces the infectivity by 80–96% [14-16]. In the baseline scenario with no PrEP, we assumed that individuals are tested at a rate that allows the modelled CD4^+^ T-cell count distribution at diagnosis to match the CD4^+^ T-cell count distribution at diagnosis in 2015 in the Germany among newly diagnosed MSM.

**Table:** Key model parameters and costs, cost-effectiveness analysis of pre-exposure prophylaxis for HIV prevention in Germany

Description	Estimate or range^a^	Reference
Model parameters		
Duration of disease stages		
Acute stage	10–16 weeks	[38]
CD4^+^ T-cell count 350–500 cells/µL	2.9–3.1 years	[39]
CD4^+^ T-cell count 200–349 cells/µL	3.6–3.9 years	[39]
CD4^+^ T-cell count < 200 cells/µL	13–25 months	[39]
Infectivity per partnership transmissibility per year		
Acute stage	0.030–0·61	[13]; Model calibration
Chronic stage	0.027–0·21	[13]; Model calibration
AIDS stage	0.008–0·27	[13]; Model calibration
On Treatment	80–96% reduction in transmissibility compared with chronic stage	[14-16]
Proportion of people in sexual risk groups		
Highest	6–15%	Model calibration, the sum of the four groups was equal to 100%
2nd	10–45%	
3rd	10–45%	
Lowest	4–70%	
Number of partners per year in each sexual risk group		
Highest	> 30–148	Model calibration
2nd	> 5–30	
3rd	> 0.5–5	
Lowest	0.02–0.5	
Mortality rates per year		
Population	0.0155	[40]
Chronic HIV stage	0.114	[40]
AIDS stage	0.648	[40]
On treatment	0.0184	[40]
Primary cost parameters (costs listed are in 2015 euros) from a healthcare payer perspective		
Yearly cost of ART and patient monitoring ^b^	EUR 17,016	Local data
Yearly cost of PrEP ^c^	EUR 824	Local data

AIDS: acquired immunodeficiency syndrome; ART: antiretroviral drug treatment; HIV: human immunodeficiency virus; PrEP: pre-exposure prophylaxis.

^a^ All ranges were uniformly distributed.

^b^ Averaged across regimens by percentage of patients on different regimens. Includes costs of ART, clinic visits, biannual viral load and yearly CD4^+^ T-cell count.

^c^ Includes cost of PrEP, patient monitoring, HIV testing every 3 months, testing for sexually transmitted diseases (including hepatitis C, syphilis, chlamydia and gonorrhea) every 6 months.

We calibrated our model to the historic HIV epidemic based on the estimated population size of German MSM [17], the number of MSM diagnosed with HIV, the percentage diagnosed with a CD4^+^ T-cell count greater than 500 cells per μL, the percentage diagnosed with a CD4^+^ T-cell count less than 200 cells per μL, and the estimated number of MSM living with HIV in Germany [11] (Supplementary Table S1). The model included four different sexual risk groups with different levels of sexual activity based on the annual number of new sexual partners. The annual number of new sexual partners ranged from > 30 to 148 in the group with highest sexual activity, > 5 to 30 in the group with second highest sexual activity, one to five partners per 2 years in the third group and finally less than one new partner every 2 years in the group with lowest level of sexual activity. Monte Carlo filtering techniques using wide ranges of sexual activity in the different groups allowed us to identify which sexual risk group combinations resulted in the appropriately calibrated HIV epidemic. We accepted 862 of 1 million simulations that matched the German HIV epidemic among MSM. All results are reported as median and interquartile range (IQR) of the accepted simulations.

PrEP was targeted at 30% of the two most sexually active groups in the model, which are defined as high-risk, or at a median of 60,000 MSM at high risk of infection. PrEP was initiated in 2017 and scaled up over 2 years. The median duration of PrEP use was assumed to be 5 years or until HIV diagnosis. Individuals using PrEP were assumed to receive an HIV test every 3 months. In our model, it was assumed that infected individuals start antiretroviral drug treatment immediately after diagnosis as recommended in the German HIV treatment guidelines [18].

### Cost-effectiveness and budget effect of PrEP

The cost-effectiveness was calculated from the healthcare payer perspective, assuming that PrEP is reimbursed. Each compartment in our deterministic model was assigned a cost and quality-adjusted life years (QALY). The costs in our analysis were based on micro-costing of the unit costs of each component of resource use [19] by 362 German HIV-infected MSM that were included in 17 centres as reported elsewhere [20]. The values have been adjusted to reflect 2016 values using the harmonised German general consumer price index [21]. The cost components that we included were costs of the antiretroviral drugs, direct healthcare costs and indirect healthcare costs. The costs of antiretroviral drugs were based on the on the costs of the weighted mean of the antiretroviral drug regimens used in Germany [22] (Supplementary Table S3). Direct healthcare costs included outpatient visits to an HIV specialist and to other medical specialists, hospitalisation and rehabilitation (Supplementary Table S4). Indirect healthcare costs included home care, domestic help, travel costs and sick leave (included because health insurance in Germany pays sick leave exceeding 6 weeks, while the costs of the first 6 weeks were not included as these are paid by the employer) (Supplementary Table S4). The costs that we included for PrEP were the price of antiretroviral drugs used as PrEP, the costs of visiting a physician who prescribes PrEP and the costs of monitoring side effects of tenofovir (e.g. creatinine) and monitoring sexually transmitted infections (including HIV, syphilis, hepatitis C and other bacterial sexually transmitted infections) (Supplementary Table S2).

We calculated the budget effect and QALY gained over a 40-year period, which was the estimated lifespan between the average age at HIV diagnosis and the male life expectancy in Germany. Costs and QALY were discounted at 3% per year [23]. Cost-effectiveness was calculated as the QALY gained divided by the difference in costs comparing PrEP to no PrEP. The QALY weights can be found in Supplementary Table S5. The total budget effect was the difference in costs comparing PrEP to no PrEP. 

### Sensitivity analysis

We performed a univariate sensitivity analysis of the cost-effectiveness and budget effect of introducing PrEP compared with not using PrEP. Six key input variables were considered to assess the sensitivity of our model. The yearly cost of antiretroviral drug treatment was ranged between the price of EUR 17,000 and EUR 3,500 (the lower range of EUR 3,500 reflected a, assumed reduction of the annual antiretroviral drug costs of EUR 15,000 by 90% plus EUR 2,000 for direct and indirect healthcare costs; Table and Supplementary Table S4). We also varied the annual price of PrEP between EUR 821 for generic PrEP (Table) and EUR 8,123 for tenofovir alafenamide fumarate (TAF), a different prodrug of tenofovir. The effectiveness of PrEP was ranged between 45%, the lowest efficacy of PrEP reported among MSM [3], and 95%. In the sensitivity analysis, the coverage of PrEP among MSM at high risk for HIV infection was varied between 10% and 65% [7]. We also varied the coverage of PrEP among MSM at low risk of infection from 0% to 30%. Finally, we did a multivariate sensitivity analysis using recursive partitioning to determine the most influential independent parameter on the budget effect of PrEP (Supplement, pp 8–9).

## Results

### Impact of PrEP on the HIV epidemic

**Figure 1:**
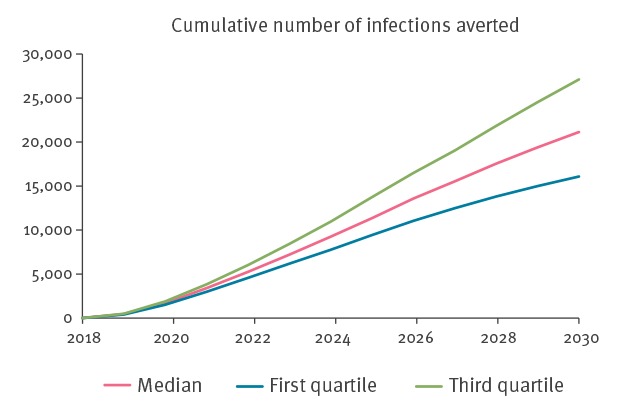
Short-term epidemiological impact of PrEP on HIV prevention, modelled cost-effectiveness, Germany, 2018–2030

HIV: human immunodeficiency virus; MSM: men who have sex with men; PrEP: pre-exposure-prophylaxis.

At 85% effectiveness, PrEP could prevent 21,000 (IQR: 16,000–27,000) new infections in the short run (after 2 years scale-up and 10 years full implementation), if targeted to 30% of MSM at high risk of HIV infection (Figure 1).

Short-term (2018–2030) epidemiological impact on the German HIV epidemic of PrEP targeted at 60,000 MSM at high risk of HIV infection and at 85% effectiveness in reducing the risk for HIV infection. The lines represent the predicted number of HIV infections that will be averted based on 862 simulations that were calibrated to the historic German HIV epidemic. The red line is the median value, and the interquartile ranges are represented by the blue line (first quartile) and the green line (third quartile).

At 85% effectiveness and 3% yearly QALY discounting, the introduction of PrEP was predicted to result in a median gain of 200,000 QALY (IQR: 150,000–270,000) over 40 years. Ranging the yearly QALY discounting rate resulted in a median gain of 290,000 QALY (IQR: 210,000–380,000) at a yearly rate of 1.5% and 150,000 QALY (IQR: 110,000–200,000) at a yearly rate of 4.5%.

### Budget effect of PrEP

Treatment and monitoring of patients infected with HIV was predicted at a discounted cost of EUR 36.2 billion (IQR: 32.4 billion–40.4 billion) over the coming 40 years in Germany. At 85% effectiveness, introduction of PrEP at a generic price was predicted to be cost-saving and to reduce the discounted costs of HIV care by EUR 5.1 billion (IQR: 3.5 billion–6.9 billion) over a 40-year time period. We did not calculate a cost-effectiveness ratio as introduction of PrEP would result in substantial health gains (measured as a reduction of infections and as a gain of QALY) at a lower cost compared with the situation without PrEP.

**Figure 2:**
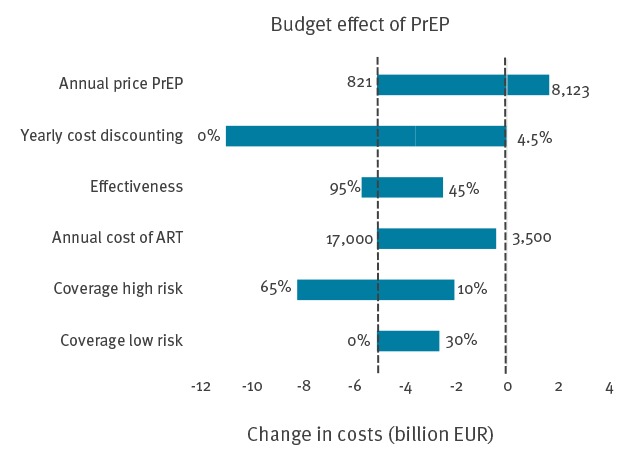
One-way sensitivity analysis of the modelled change in costs of HIV care and prevention over 40 years, comparing PrEP use with no PrEP, Germany, 2018–2058

ART: antiretroviral drug treatment; MSM: men who have sex with men; HIV: human immunodeficiency virus; PrEP: pre-exposure-prophylaxis.

Figure 2 shows the one-way sensitivity analysis about the impact of six key parameters on the budget effect. Introduction of PrEP was only predicted to result in increased costs if a branded version of TAF was used as PrEP. The increased discounted costs of branded TAF were EUR 1.66 billion or a cost-effectiveness ratio of EUR 8,500 per QALY. Although the budget effect was also sensitive to the price of antiretroviral drug treatment, introduction of PrEP was predicted to remain cost-saving even at the strongest reduction in the costs of antiretroviral drug treatment by 90% (to EUR 3,500/year). The sensitivity analysis also showed that PrEP would save at least EUR 2 billion in discounted costs for the most pessimistic ranges of the other key parameters including an increased yearly discounting rate of 4.5%, a reduced effectiveness of PrEP of 45% in preventing new infections, a reduction to 10% of the proportion of high-risk MSM that receive PrEP and a proportion of 30% of MSM at low risk of infection who receive PrEP (Figure 2).

The annual price of PrEP ranged between EUR 821 for generic PrEP and EUR 8,123 when using branded tenofovir alafenamide fumarate. The annual cost for ART was ranged between the current price of EUR 17,000 (which included antiretroviral drugs and direct and indirect health care costs) and EUR 3,500 which would be the cost if the price of antiretroviral drugs were reduced by 90% and the indirect and direct health care costs remained the same at EUR 2,000 per year. The coverage of PrEP in low-risk individuals (or the proportion of individuals in the two groups with lowest sexual activity) ranged between 0 and 30%. The coverage in high-risk MSM (or the proportion of MSM in the two groups with highest sexual activity) ranged between 10% and 65% [7].

The dashed line represents the budget effect of introducing PrEP targeted to 30% of individuals at the highest level of sexual activity and at an effectiveness of PrEP of 85%.

The annual price of PrEP ranged between EUR 821 for generic PrEP and EUR 8,123 assuming the use of branded TAF. The annual cost for antiretroviral drug treatment was ranged between EUR 17,000 and EUR 3,500. We ranged the coverage of PrEP in low-risk individuals (or the proportion of individuals in the two groups with lowest sexual activity that will use PrEP) between 0 and 30%. The coverage in high-risk MSM (or the proportion of MSM in the two groups with highest sexual activity) was ranged between 10% and 65% [7].

**Figure 3:**
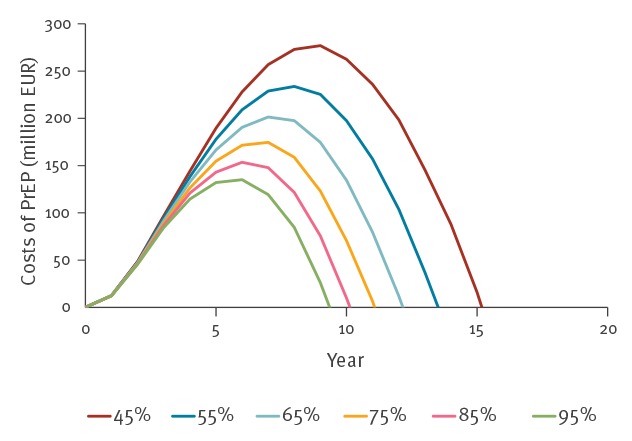
Short-term cumulative costs of a PrEP programme, modelled cost-effectiveness, Germany, 2018–2058

HIV: human immunodeficiency virus; MSM: men who have sex with men; PrEP: pre-exposure-prophylaxis.

Although PrEP was predicted to be cost-saving in the long term, introduction of PrEP could in the first 10 years result in increased expenditure of HIV-related costs ranging between a discounted EUR 135 million and EUR 275 million for an effectiveness of 95% and 45%, respectively (Figure 3). The break-even point, calculated as the number of years when the cumulative savings from averted HIV infections begin to exceed the costs of a PrEP programme [24], was reached after 10 years. In Figure 3, we also show that the short-term costs and the break-even point were predicted to depend on the effectiveness of PrEP in daily practice. At an effectiveness of 45%, the break-even point was reached after 45 years. Similarly, at an effectiveness of 95%, PrEP cost at most EUR 135 million and the break-even point was reached after 9 years.

The lines in the graph represent the change in the discounted cumulative costs, defined as the difference between a scenario in which PrEP is available and PrEP is not available, at different effectiveness of tenofovir disoproxil fumarate and emtricitabine in reducing the risk for HIV infection. PrEP is assumed to start in 2018 (year 0 on the horizontal axis). Data are only presented for those years after introduction in which the costs of PrEP exceeded the costs of infections that will be averted. The break-even point is represented by the point where the lines cross the axis representing the number of years after introduction of PrEP.

**Figure 4:**
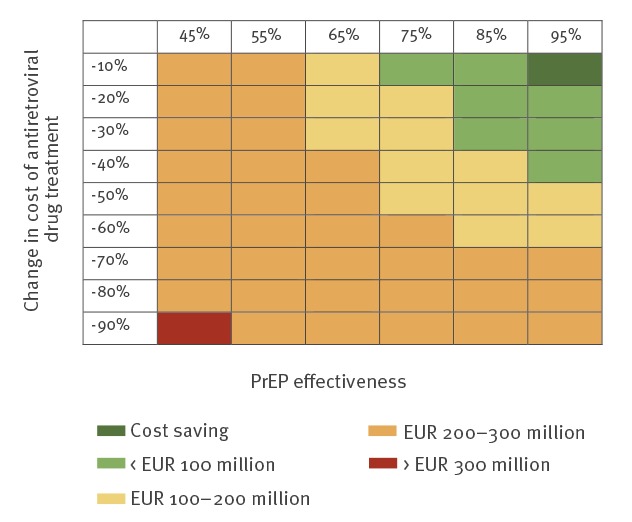
Cumulative costs (annually discounted at 3%) during the first 10 years after introduction of PrEP, stratified by effectiveness of PrEP and reduction in costs of antiretroviral drug treatment, cost-effectiveness model, Germany, 2018–2029

**Figure 5:**
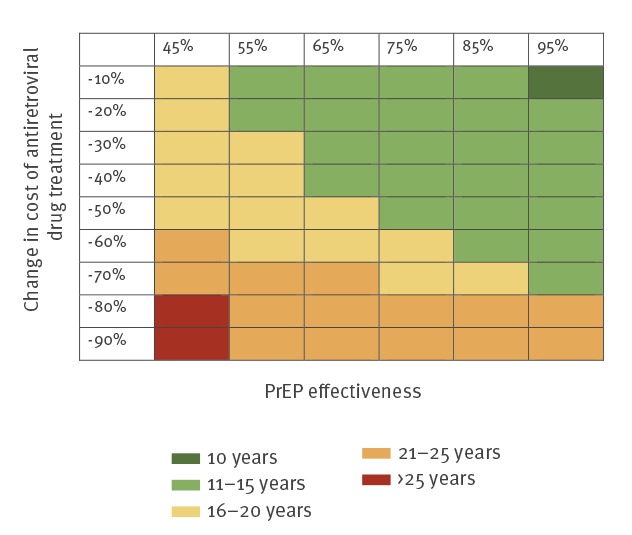
Minimum of years to reach break-even point in which the cumulative discounted (at an annual rate of 3%) costs of averted HIV infections exceed the costs of a PrEP programme, cost-effectiveness model, Germany, from 2018 onwards

HIV: human immunodeficiency virus; PrEP: pre-exposure prophylaxis.

Because the one-way sensitivity analysis showed that a reduction in the future costs of antiretroviral drugs would have a profound impact on the budget effect of PrEP, we further explored the short-term economic costs and the break-even point in a two-way sensitivity analysis in which we ranged the costs of antiretroviral drugs and the effectiveness of PrEP (Figures 4 and 5). The cumulative costs after 10 years were predicted to be higher at a lower effectiveness of PrEP in reducing the risk of HIV infection. Higher cumulative costs were also found at the strongest reductions in the price of antiretroviral drug treatment. The cumulative costs were predicted to remain below EUR 300 million after 10 years, except when PrEP had an effectiveness of 45% and the price of antiretroviral drugs was reduced by 90% (Figure 4). Figure 5 shows that the break-even point also strongly depended on the effectiveness of PrEP and a possible reduction in the price of antiretroviral drug treatment. If PrEP is at least 45% effective, introduction of PrEP is predicted to become cost-saving within 20 years at a reduction in the price of antiretroviral drug treatment of at most 50%. At a lower effectiveness of PrEP and at price reductions exceeding 50%, the break-even point occurred later. Reductions in the price of antiretroviral drug treatment by more than 50% move the break-even point to between 20 and 25 years after introduction of PrEP. A strong reduction in the price of antiretroviral drug treatment of more than 80% at an effectiveness of PrEP of 45% resulted in a break-even point reached after more than 25 years.

PrEP: pre-exposure prophylaxis.

Costs are shown as million EUR. The undiscounted costs can be found in Supplementary Table S6.

The analysis is stratified by the effectiveness of PrEP in reducing the risk of HIV infection and by the future costs of antiretroviral drug treatment compared with the current costs. PrEP is assumed to be initiated in 2018. The break-even point for the undiscounted costs can be found in Supplementary Table S7.

## Discussion

Our model predicts that the use of PrEP for HIV prevention among MSM in Germany is cost-saving over a 40-year time period and leads to substantial health benefits in that it reduces the number of new HIV infections. Although PrEP is predicted to be cost-saving over a prolonged period of time, the overall HIV-related costs can increase in the first 10 years after introduction.

Our modelling results and cost-effectiveness analysis are in agreement with recent European modelling studies on the economic impact of PrEP in the Netherlands [9] and in the United Kingdom (UK) [24,25], countries that, similar to Germany, have an HIV epidemic concentrated among MSM. In the Netherlands, the introduction of generic versions of tenofovir and emtricitabine has reduced the price of PrEP by 90% [26]. At this price level and targeted to 10% of high-risk MSM, generic PrEP is predicted to be cost-saving in the Netherlands over a 40-year time period [9]. Similarly, Cambiano et al. report that PrEP targeted to high-risk MSM in the UK would be cost-saving within a 40-year time period and that a reduction of the cost of antiretroviral drugs (including drugs used as PrEP) would substantially shorten the time for cost savings to be realised [25]. Ong et al. found that PrEP targeted to high-risk MSM visiting genito-urinary medicine clinics will be cost-saving over a 10-year time period if the price of generic PrEP is reduced as strongly as in Germany [24]. Contrary to our and others models [9,25], however, Ong et al. used a static model [24] which cannot assess the preventive benefits of PrEP at population level and which may therefore underestimate the cost-effectiveness of PrEP.

The number of MSM at high risk of HIV infection who will use PrEP is a key parameter for the epidemiological and economic impact of a PrEP programme. Our modelling study shows that if a larger proportion of individuals at high risk of infection are using PrEP, more HIV infections will be prevented and more costs will be saved in the long term. In the first decade after introduction, however, a higher uptake of PrEP will result in higher economic expenses because in the short term, costs of PrEP will exceed the cumulative savings from averted HIV infections. Although the future uptake of PrEP in Germany is unknown, a web-based questionnaire found that 65% of participating HIV-negative MSM at risk of infection were willing to use PrEP [27].

Two independent randomised controlled trials showed that PrEP can prevent ca 85% of new infections if used daily [4] or if used on demand, where a high-risk individual uses PrEP 2–24 h before sexual contact, followed by additional dosages 24 h and 48 h after the last sexual contact [5]. Cost-effectiveness studies showed that on-demand PrEP is more cost-effective than daily use as such a dosing scheme uses fewer tenofovir and emtricitabine tablets [9]. A PrEP demonstration project in the Netherlands, however, reported that almost three times more eligible MSM prefer the more expensive daily PrEP than on-demand PrEP [28]. In our analysis, we only included daily use of PrEP and found this approach to be cost-saving. In daily practice, some individuals will prefer an on-demand dosing scheme [28], which will reduce the costs of PrEP.

Our analyses predict that a reduction in the price of antiretroviral drug treatment will increase the time for cost savings to be realised. Importantly, the cost of antiretroviral drug treatment may strongly decrease as generic versions of TDF and emtricitabine cannot only be used as PrEP, but also in first-line treatment of HIV. We argue, however, that the costs of antiretroviral drug treatment in Germany will remain high. Firstly, only one tenofovir prodrug, TDF, has become available as a generic product. The patent of another prodrug of tenofovir, TAF, will not expire in the coming years. In Germany, TAF is preferred over TDF [18] as TAF is associated with a lower level of renal and bone mineral toxicity [29]. Indeed, a strong shift towards TAF based-regimens has been observed since 2016 across Germany [30]. Secondly, TDF and emtricitabine in HIV therapy have to be combined with a third drug. The most widely used third drug class are integrase strand transfer inhibitors such as dolutegravir or elvitegravir which are still protected by a patent [31] and will therefore remain costly in the coming years.

Our mathematical modelling analysis has several strengths. Firstly, we calibrated our model to the well-described German HIV epidemic [11], which allowed us to make accurate epidemic predictions. Secondly, we used micro-costing to obtain the overall costs of HIV. Micro-costing, involving the detailed estimation of the unit costs of each component of resource use [19], is time-consuming and not frequently performed. Nonetheless, micro-costing is considered the most precise level of healthcare costing [19]. The final strength of our study is that we considered the population benefit of PrEP using an HIV transmission model [9,25,32].

Our study has several limitations. Firstly, we did not consider risk compensation, defined as an increase in sexual risk behaviour in response to the use of PrEP, which can result in increased rates of bacterial sexually transmitted infections and hepatitis C virus infections. Although recent trials have not shown a substantial increase in risk behaviour [4], increases in bacterial sexually transmitted infections in those on PrEP in real-world settings [33]. In addition, infections with hepatitis C virus have been reported in a few HIV-negative MSM that use PrEP [34]. Introduction of PrEP may therefore result in increased costs owing to higher rates of sexually transmitted infections. PrEP is, however, expected to remain cost-saving because bacterial sexually transmitted infections are treated with generic antibiotics that are cheap. Conversely, substantial increases in the incidence of hepatitis C, which require treatment with expensive direct-acting antiviral drugs [35], can result in a less favourable cost-effectiveness of PrEP. Secondly, previous modelling studies showed that PrEP is only cost-effective when targeted to individuals at a high risk of HIV infection [36]. It cannot be ruled out that MSM who are at low risk of infection perceive themselves to be at high risk and therefore start using PrEP. Our sensitivity analysis, however, predicted that introduction of PrEP remains cost-saving even when a substantial proportion of MSM at low risk of infection start using PrEP. Thirdly, we assumed that the German HIV epidemic among MSM will remain at a similar level as it is today if PrEP is not introduced. The number of new HIV diagnoses among MSM in the Netherlands [10] and the UK [37] has declined in recent years, which has been ascribed to increased testing followed by immediate treatment in those testing positive. A similar decline in Germany would decrease the costs that can be saved and prolong the time for cost-savings to be realised.

## Conclusion

Introduction of PrEP in Germany can reduce the HIV epidemic among MSM in a cost-saving manner. PrEP is predicted to remain cost-saving even when generic versions of antiretroviral drug treatment become available. Introduction of PrEP will, however, require short-term financial investments which are predicted to result in substantial cost-savings after a period of at least 10 years.

## Supplementary Data

297000 Supplement
