# Supplementary material for: Cost-effectiveness and budget effect of pre-exposure prophylaxis for HIV-1 prevention in Germany from 2018 to 2058
Source: Euro Surveill. 2019 Feb 14;24(7):1800398. doi: 10.2807/1560-7917.ES.2019.24.7.1800398 (PMC6381659; doi:10.2807/1560-7917.ES.2019.24.7.1800398)
Supplement: Supplement [file 18-00398_Supplement.pdf]

Supplement

Epidemiological impact and budget effect of pre-exposure prophylaxis for HIV-1 prevention in Germany from 2018 to 2058

This supplementary material is hosted by *Eurosurveillance* as supporting information alongside the article Epidemiological impact and budget effect of pre-exposure prophylaxis for HIV-1 prevention in Germany from 2018 to 2058 on behalf of the authors who remain responsible for the accuracy and appropriateness of the content. The same standards for ethics, copyright, attributions and permissions as for the article apply. *Eurosurveillance* is not responsible for the maintenance of any links or email addresses provided therein."

Introduction

This document provides additional information on the mathematical model, calibration and multivariate sensitivity analysis of study on the cost-effectiveness and budget effect of preexposure prophylaxis (PrEP) in Germany.

Mathematical transmission model

This study includes a compartmental deterministic mathematical transmission model that was developed for the HIV epidemic among men-who-have-sex-with-men (MSM) in the Netherlands[1, 2] and that was adapted to the HIV epidemic among MSM in Germany. The schematic representation of the model is presented in figure S1.

Figure S1 – *Schematic representation of the compartmental deterministic model (the mathematical equations can be found on pages 3-4 of the supplement). The state variables used in the equations are shown between brackets.*
The parameters used in the equations are added next to the arrows that indicate the rate of change between the different compartments of the model. We assume that individuals can only start using PrEP when they are not infected with HIV. Individuals can become infected with HIV despite the use of PrEP.The arrows for infected (but not using PrEP) go through the boxes of individuals using PrEP that have an unrecognized HIV infection to the boxes of individuals that have been diagnosed but who do not use treatment (yet).

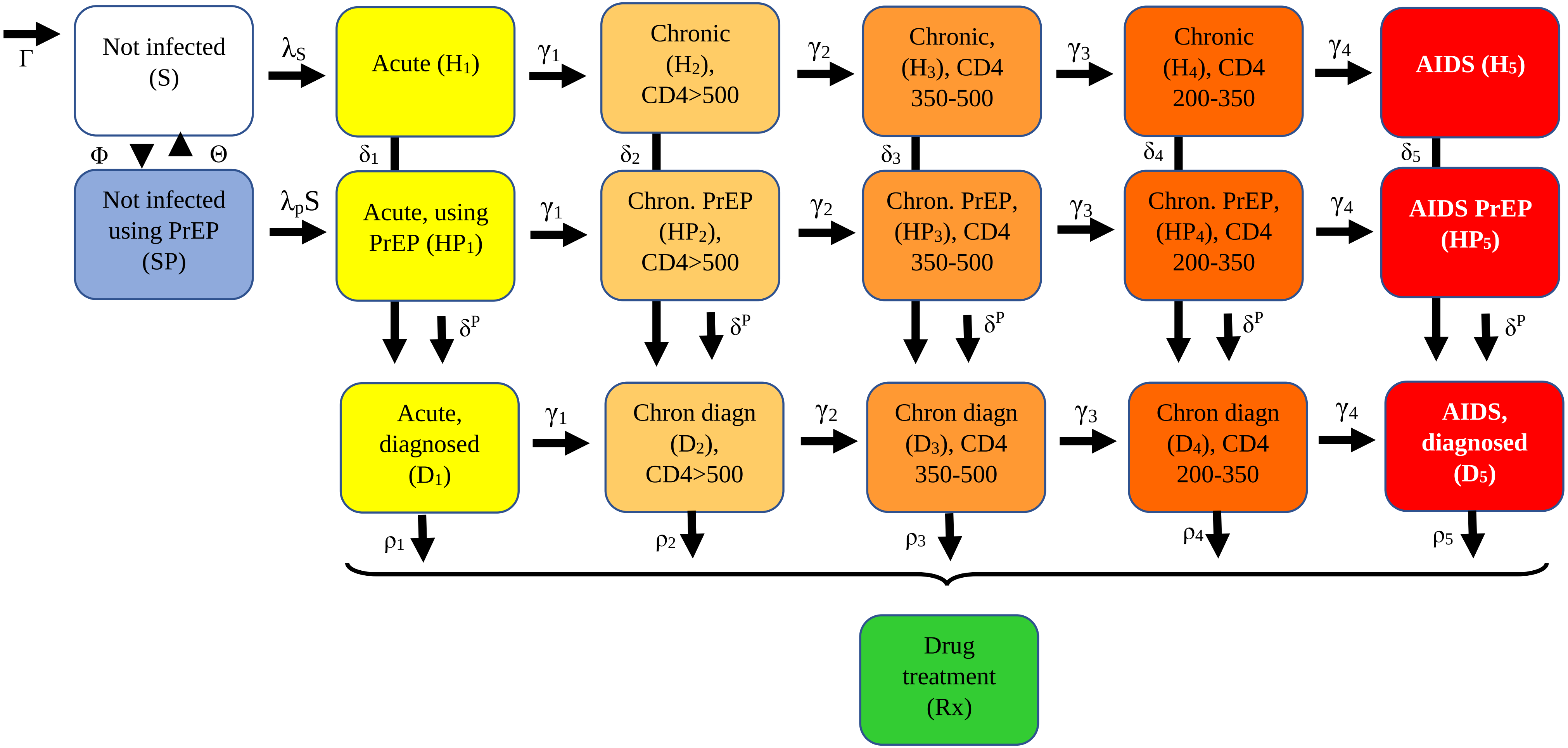

Equations of the transmission model

The model consists of 15 ordinary differential equations, including two equations that describe individuals that are not infected with HIV that use PrEP or do not use PrEP, ten equations that describe disease progression, two equations that describe the force of infection (or the rate by which individuals become infected)[3] and one equation that describes mixing between individuals of different risk groups[3, 4]. The equations are summarized below.

Ordinary differential equations for people not infected with HIV

Individuals not infected with HIV are sub-divided into individuals that are not on PrEP, denoted as S (susceptible individuals modelled using equation 1) and individuals using PrEP denoted as SP (suspeptible individuals using PrEP, modelled using equation 2). The sexual activity classes are defined in the equations in four groups *a* ranging from the group with the highest sexual activity (*a* = 1) to the group with the lowest sexual activity (*a* = 4). Susceptible individuals have a mortality rate μ. Individuals that become sexually active enter the group of susceptible individuals not using PrEP at a rate Γ times the proportion π_a of each sexual activity group *a.* Values for the parameter Γ have been calibrated to the German MSM population[5] (Table S1). Susceptible individuals can start PrEP at a rate Θ_a and discontinue PrEP at a rate Φ_a (the values Θ_a and Φ_a for the lowest sexual activity groups *a=3* and *a=4* were set to zero as we assumed that PrEP will only be used for MSM at high risk for HIV infection). The force of infection, denoted as λ_a for individuals not using PrEP (modelled using equation 13) and $\lambda^P{}_a$ for individuals using PrEP (equation 14), represents the rate by which individuals become infected with HIV.

$$S_a' = \Gamma.\pi_a - S_a(\lambda_a + \Theta_a + \mu) + SP_a.\Phi_a \quad (1)$$

$$SP_a' = S_a.\Theta_a - SP_a(\lambda^P{}_a + \Phi_a + \mu) \quad (2)$$

Equations for undiagnosed HIV infected individuals

Individuals that become infected with HIV but that have not been diagnosed yet (denoted with *H* in equations 3 through 5), progress through five stages of infection that are denoted by the parameter σ. These stages of infection are the acute stage (σ = 1, modelled using equation 3), the chronic stages (σ = 2 if the CD4 cell count is > 500 cells/µl, σ = 3 for a CD4 between 350 and 500 cells/µl and σ = 4 for a CD4 between 200 and 350 cells/µl, all modelled using equation 4) and the AIDS stage (σ = 5, modelled using equation 5). Individuals progress through infection at a rate γ_σ that depends on the stage of infection σ[6]. Individuals are diagnosed at a rate δ_σ that has been calibrated to the proportion of MSM that are diagnosed at a particular CD4 threshold as reported by the Robert Koch Institute. The mortality rate depends on the stage of infection (denoted as μ_σ).

$$H_{\sigma,a}' = \lambda_a.S_a - H_{\sigma,a}(\delta_\sigma + \gamma_\sigma + \mu_\sigma)\text{..... for } \sigma = 1 \quad (3)$$

$$H_{\sigma,a}' = H_{\sigma-1,a}.\gamma_{\sigma-1} - H_{\sigma,a}(\delta_\sigma + \gamma_\sigma + \mu_\sigma)\text{..... for } \sigma = 2, 3, 4 \quad (4)$$

$$H_{\sigma,a}' = H_{\sigma-1,a}.\gamma_{\sigma-1} - H_{\sigma,a}(\delta_\sigma + \mu_\sigma)\text{..........for } \sigma = 5 \quad (5)$$

Equations for HIV infected undiagnosed individuals using PrEP

The equations used to model individuals that become infected with HIV despite the use of PrEP (denoted as *HP* in equations 6 through 8), are comparable to the equations used to model HIV-infected individuals that do not use PrEP (equations 3, 4 and 5). Individuals using PrEP are assumed to be tested for HIV every six months at a rate (denoted as δ^P).

$$HP_{\sigma,a}' = \lambda^P{}_a.SP_a - HP_{\sigma,a}(\delta^P + \gamma_\sigma + \mu_\sigma)\text{..... for } \sigma = 1 \quad (6)$$

$$HP_{\sigma,a}' = HP_{\sigma-1,a}.\gamma_{\sigma-1} - HP_{\sigma,a}(\delta^P + \gamma_\sigma + \mu_\sigma)\text{..... for } \sigma = 2, 3, 4 \quad (7)$$

$$HP_{\sigma,a}' = HP_{\sigma-1,a}.\gamma_{\sigma-1} - HP_{\sigma,a}(\delta^P + \mu_\sigma)\text{..........for } \sigma = 5 \quad (8)$$

Equations for HIV untreated infected individuals that have been diagnosed

Individuals that are diagnosed with HIV but that are not treated with antiretroviral drugs (yet) are denoted as *D* (equations 9 through 11). Diagnosed individuals start treatment at a rate ρ_σ. The rate ρ_σ depends on the stage of infection σ to reflect past changes in the CD4 cell count at which treatment was initiated. Per recent treatment guidelines, antiretroviral drugs are started irrespective of the stage of infection as of 2017 when we assumed that PrEP became available in Germany.

$$D_{\sigma,a}' = H_{\sigma,a}.\delta_\sigma. + HP_{\sigma,a}.\delta^P - D_{\sigma,a}(\gamma_\sigma + \rho_\sigma + \mu_\sigma)\text{ ..... for } \sigma = 1 \quad (9)$$

$$D_{\sigma,a}' = H_{\sigma,a}.\ \delta_\sigma.+ HP_{\sigma,a}.\delta^P + \gamma_{\sigma-1}.D_{\sigma-1,a} - D_{\sigma,a}(\gamma_\sigma + \rho_\sigma + \mu_\sigma)\text{ ..... for } \sigma = 2, 3, 4 \quad (10)$$

$$D_{\sigma,a}' = H_{\sigma,a}.\ \delta_{\sigma}. + HP_{\sigma,a}.\delta^{P} + \gamma_{\sigma-1}.D_{\sigma-1,a} - D_{\sigma,a}(\rho_{\sigma} + \mu_{\sigma}) \ \ldots\ldots \text{ for } \sigma = 5 \quad (11)$$

Equations for individuals using antiretroviral drug treatment

Individuals that use antiretroviral drugs are represented by the state variable *Rx* (equation 12). People treated with antiretroviral drug treatment are assumed to have the same mortality as the general population[7].

$$Rx_a' = \sum_{\sigma=1}^{5} \rho_{\sigma} D_{\sigma,a} - Rx_a \mu \quad (12)$$

Equations for the force of infection

The force of infection is modelled using equation 13 for individuals not using PrEP (λ_a), and using equation 14 for individuals using PrEP ($\lambda^P{}_a$). The force of infection depends on the rate of sexual partner change (c_a) for individuals with sexual activity *a*, and the probability by which these individuals form a sexual relationship with an individual with sexual activity *i* as determined by the mixing matrix $M_{a,i}$ (equation 15). The rate of infection also depends on the infectivity of the different stages of infection represented. The model uses three different parameters of infectivity that all depend on the stage of infection σ, including β_σ for untreated HIV infected individuals that do not use PrEP, $\beta_\sigma{}^P$ for individuals using PrEP and β_{Rx} for individuals using antiretroviral drug treatment.

$$\lambda_a = c_a \sum_{i=1}^{4} \frac{M_{a,i}}{N_i} \left(\sum_{\sigma=1}^{5} \beta_\sigma H_{\sigma,i} + \sum_{\sigma=1}^{5} \beta_\sigma{}^P HP_{\sigma,i} + \sum_{\sigma=1}^{5} \beta_\sigma D_{\sigma,i} + \beta_{Rx} Rx_i\right) \quad (13)$$

$$\lambda^P{}_a = c_a \sum_{i=1}^{4} \frac{M_{a,i}}{N_i} \left(\sum_{\sigma=1}^{5} \beta_\sigma{}^P H_{\sigma,i} + \sum_{\sigma=1}^{5} \beta_\sigma{}^P HP_{\sigma,i} + \sum_{\sigma=1}^{5} \beta_\sigma{}^P D_{\sigma,i} + \beta_{Rx}{}^P Rx_i\right) \quad (14)$$

Mixing matrix

$M_{a,j}$ is a mixing matrix in which the elements *a,j* are the probability that an individual in sexual activity class *a* forms a sexual partnership with an individuals with sexual activity *j*. The mixing matrix includes a factor ε which denotes the degree of assortative mixing, and $\delta_{a,j}$ denotes Kronecker delta which is equal to zero if individuals are in the same sexual activity class or equal to one if the individuals are in different sexual activity classes[4].

$$M_{a,i} = \varepsilon.\delta_{a,i} + (1 - \varepsilon)\ c_a \frac{N_i}{\sum_{a=1}^{4} c_a n_a} \quad (15)$$

Model calibration

The model has been calibrated to the historic HIV epidemic based on: the estimated German MSM population size[5], number of MSM diagnosed with HIV, percentage diagnosed with a CD4 greater than 500 cells per µl and percentage diagnosed with a CD4 cell count less than 200 cells per µl, estimated number of MSM living with HIV in Germany and the estimated number of new infections[8] (Table S1).

Table S1 *Variables used to calibrate and accept simulation using Monte Carlo filtering techniques. A total of 862 simulation were accepted (out of one million simulations run).*

Parameter used for calibration	Data in real world	Values accepted in calibration<br>Median (minimum - maximum)	Source
MSM population (15+)<br>2013<br>2014<br>2015	 850,000<br>856,000<br>866,000	 862,000 (820,000 – 900,000)<br>863,000 (820,000 – 910,000)<br>864,000 (820,000 – 920,000)	[5]
Number of new diagnosis among MSM<br>2013<br>2014<br>2015	 1728<br>1894<br>1851	 1892 (1502- 2196)<br>1972 (1653- 2397)<br>2072 (1833 – 2513)	[8-10]
Proportion diagnosed at a CD4 of<br>>500 cells/µl<br>350 – 500 cells/ µl<br>200 – 350 cells/ µl<br><200 cells/ µl	 31%<br><br><br>30%	Proportions add up to 100%<br>33% (20 – 40%)<br>22% (10 – 30%)<br>22% (10 - 30%)<br>23% (15 – 30%)	[8-10]
Number of MSM living with HIV	56,000	50,000 (45,000 – 65,000)	[8-10]

Costs

The costs are considered from the perspective of the health care payer (statutory health insurance).

Costs of PrEP

In our analysis we assumed that PrEP will be reimbursed by the German health care payer. As such we included the costs of PrEP into our analysis. The cost for PrEP comprise regular physician visits and laboratory testing during PrEP according to the German practice guidelines [11]. Unit costs for each service are derived from the Uniform Value Scale (UVS; Einheitlicher Bewertungsmaßstab)[12] for the SHI (statutory health insurance) perspective (Table S2). The annual costs for providing PrEP, including monitoring and costs of the drugs, are € 823.91.

Table S2 – *Annual costs per service from the SHI (Statutory Health Insurance) for use of Pre-exposure prophylaxis (PrEP)*

**Type of service**	**Frequency of service**	**Costs**	
		**Year 1**	**Year 2 and further**
Physician visit	Initial visit, 1 month after initiation, after that every 3 months	67.80 €	67.80 €
HIV-test	Initial visit, 1 month after initiation, after that every 3 months	16.40 €	16.40 €
Hepatitis C	Initial visit, after that once per year	9.80 €	9.80 €
Syphilis	Initial visit, 1 month after initiation, after that every 3 months	18.40 €	18.40 €
HBc-Antibodies	Initial visit	5.90 €	- €
HBs-Antigens	Initial visit	5.50 €	- €
HBs-Antibodies	Initial visit	5.50 €	- €
Hepatitis B vaccination	initial visit, after 4 and 24 weeks; every ten years	67.27 €	- €
Creatinine	Initial visit, 1 month after initiation, after that every 3 months	1.60 €	1.60 €
Liver enzymes, (ALT, ALAT)	Initial visit, 1 month after initiation, after that every 3 months	1.00 €	1.00 €
Urine culture	Initial visit, 1 month after initiation, after that every 3 months	2.00 €	2.00 €
**Total services**		**201.17 €**	**117.00 €**
Cost PrEP	Daily intake	664.82 €	
**Total cost PrEP**		**823.91** €*	

* Based on an average cost of € 159.08

Cost of HIV Treatment

We previously reported that expenditure on HIV is mainly driven by the costs of antiretroviral drugs [13] The costs of antiretroviral drugs are reimbursed by the health care payer.

The cost of antiretroviral drug treatment was based on the price of the recommended antiretroviral drug regimens for treatment of HIV in Germany[11]. The German treatment guidelines recommend to always include a backbone of two nucleoside reverse transcriptase inhibitors (NRTI) in combination with a third drug which is either a protease inhibitor (PI), a non-nucleoside reverse transcriptase inhibitor (NNRTI) or an integrase inhibitor (INI)[14]. For these ART components the mean cost per DDD (defined daily dose) was calculated based on the price of the largest package size given in the Lauer-Taxe® pharmacy price formulation[15]. In the cost calculation single-tablet preparations are distinguished from multi-tablet regimes.

To reflect current clinical practice the proportion of HIV-infected MSM receiving a given antiretroviral drug regimen is used to calculate a weighted mean ART cost. The Seroconvert-Study run by the Robert Koch-Institute (RKI) includes HIV-infected patients with a confirmed seroconversion within the three years prior to study participation [16]. Information on the antiretroviral drug regimen among MSM in this study who initiated ART in 2015 or 2016 was provided by the RKI (table S3). The annual cost per ART-regime and the weighted mean cost of ART are given in table S3. Based on these data the costs of treating a HIV-infected individual in Germany is at average € 15,010.78 per year. The main paper includes a sensitivity analysis which showed that the impact of reducing the price of antiretroviral drugs by up 90% (Table 2 and Table 3).

Table S3 *Annual costs of antiretroviral drug regimens from the SHI (Statutory Health Insurance) perspective. The costs are calculated using a backbone of two nucleoside reverse transcriptase inhibitors plus a third drug*

Third drug	Frequency	Costs
Protease inhibitor	4.9%	17,189.54 €
NNRTI	0.8%	13,180.19 €
NNRTI single-tablet	6.9%	12,207.50 €
Integrase inhibitor	32.4%	17,711.11 €
Integrase inhibitor single-tablet	55.0%	13,607.44 €
**Weighted mean**		**15,010.78 €**

We also considered costs, other than antiretroviral drugs, of treating HIV that are paid by the health care payer. These other costs physician visits, hospitalization, rehabilitation, home care, domestic help, travel cost and productivity loss that is covered by health care payer (partial or full inability to work, and sick leave after six weeks. The first six weeks of sick leave were not considered as these are paid by the employer). We based these costs on the values reported in the K3A study[13], which performed a detailed micro-costing on treatment of HIV-positive patients in 2008 in German clinical practice[13]. The values reported for 362 MSM participating in the K3A study[13] have been adjusted to reflect 2016 values using the harmonized German general consumer price index[17]. The total annual costs of treating HIV-infected MSM in Germany is € 17,015.93**,** which includes € 2,005 for non-antiretroviral drug related costs and € 15,010.78 for antiretroviral drugs (Table S4).

Table S4 *Annual costs for treating HIV, excluding the costs of antiretroviral drug treatment. Costs are presented from the SHI (Statutory Health Insurance) perspective*.

Cost component	Costs
Outpatient visits HIV specialist	269.18 €
Outpatient visits other specialists	69.91 €
Hospitalization	1,150.86 €
Rehabilitation	64.32 €
Subtotal direct cost	***1,554.27 €***
Home care	73.74 €
Domestic help	135.20 €
Travel costs	3.15 €
Sick leave	238.79 €
Subtotal indirect cost	***450.88 €***
**Total cost without antiretroviral drug treatment**	**2,005.15 €**
Antiretroviral drug treatment	15,010.78 €
**Total cost HIV treatment**	**17,015.93 €**

Utility weights

The utility weights of the quality adjusted life years (QALYs) are based on estimates from Tengs and colleagues[18]. For individuals using PrEP, we assumed a utility weight of 1[1] (Table S5).

Table S5 – *Assumed utility weighting for Quality Adjusted Life Years (QALYs), as derived from a study by Tengs and colleagues[18]*

**Status**	**Utility weight**
Not infected with HIV/using PrEP	1
CD4 > 350 cells/	0.94
CD4 cell count 200-350 cells/	0.82
AIDS stage	0.7
HIV infected using antiretroviral drug treatment	0.94

Multivariate sensitivity analysis using recursive partitioning

Recursive partitioning using the rpart library in R version 3.5.0 was used for multivariate analysis[19]. The primary endpoint in this analysis is the median budget effect for PrEP at 85% effectiveness and 30% coverage. All 32 model parameters that were varied by simulation were entered in this analysis. The N of the recursive partitioning tree represents the number of simulations that fulfill all of the given criteria for a branch in the tree. The percentage represents the proportion of simulations which are lower than the median budget effect of PrEP at current generic price with 80% effectiveness (a median saving of €5.1 billion over 40 years). The percentages highlighted in red represent branches of the tree in which 50% or more of the simulations resulted in a higher-than-median budget effect. The percentages highlighted in green represent branches of the tree in which 50% or less of the simulations resulted in a lower-than-median budget effect. Observations for which less than 90 simulations (equal to less than 10% of all simulations) were found were not included.
In our model, the proportion of MSM in in the second-highest sexual activity group (>24.8%) was the strongest predictor for lower-than-median cost savings (Figure S2).

Figure S2 – *Results of multivariate sensitivity analysis using recursive partitioning*

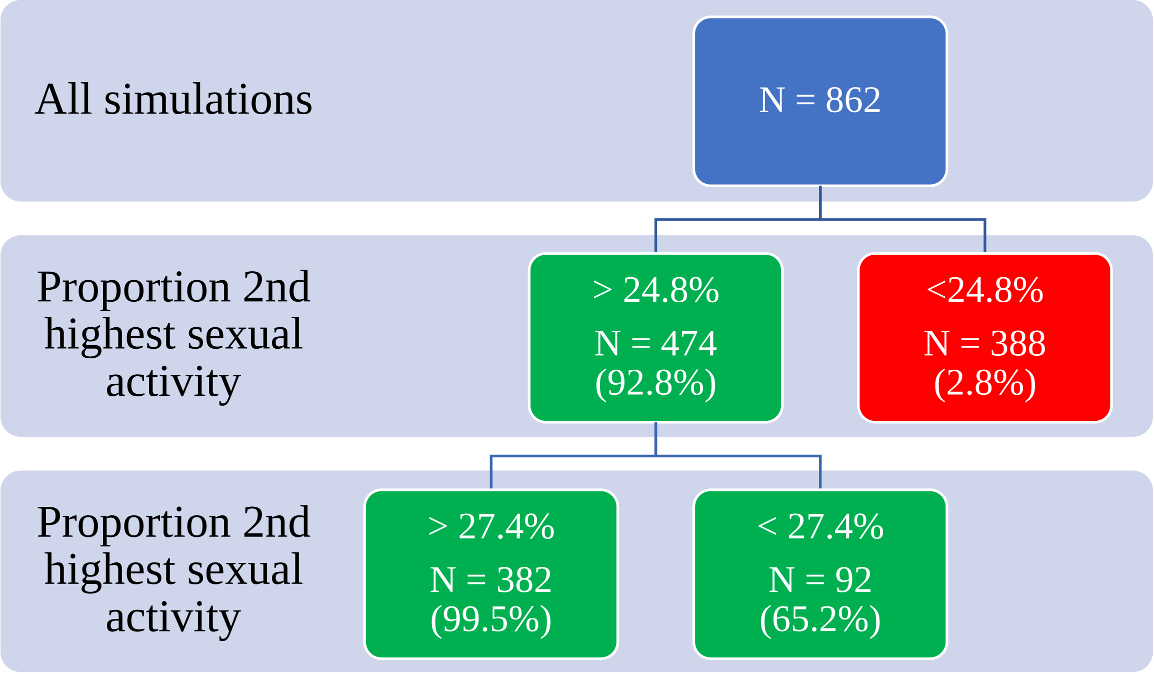

Table S6 Undiscounted cumulative costs in million Euro's during first ten years (2018-2029) after introduction of PrEP stratified by effectiveness of PrEP and by reduction in costs of antiretroviral drug treatment. The discounting costs (yearly rate of 3%) are presented in Table 2 of the main paper.

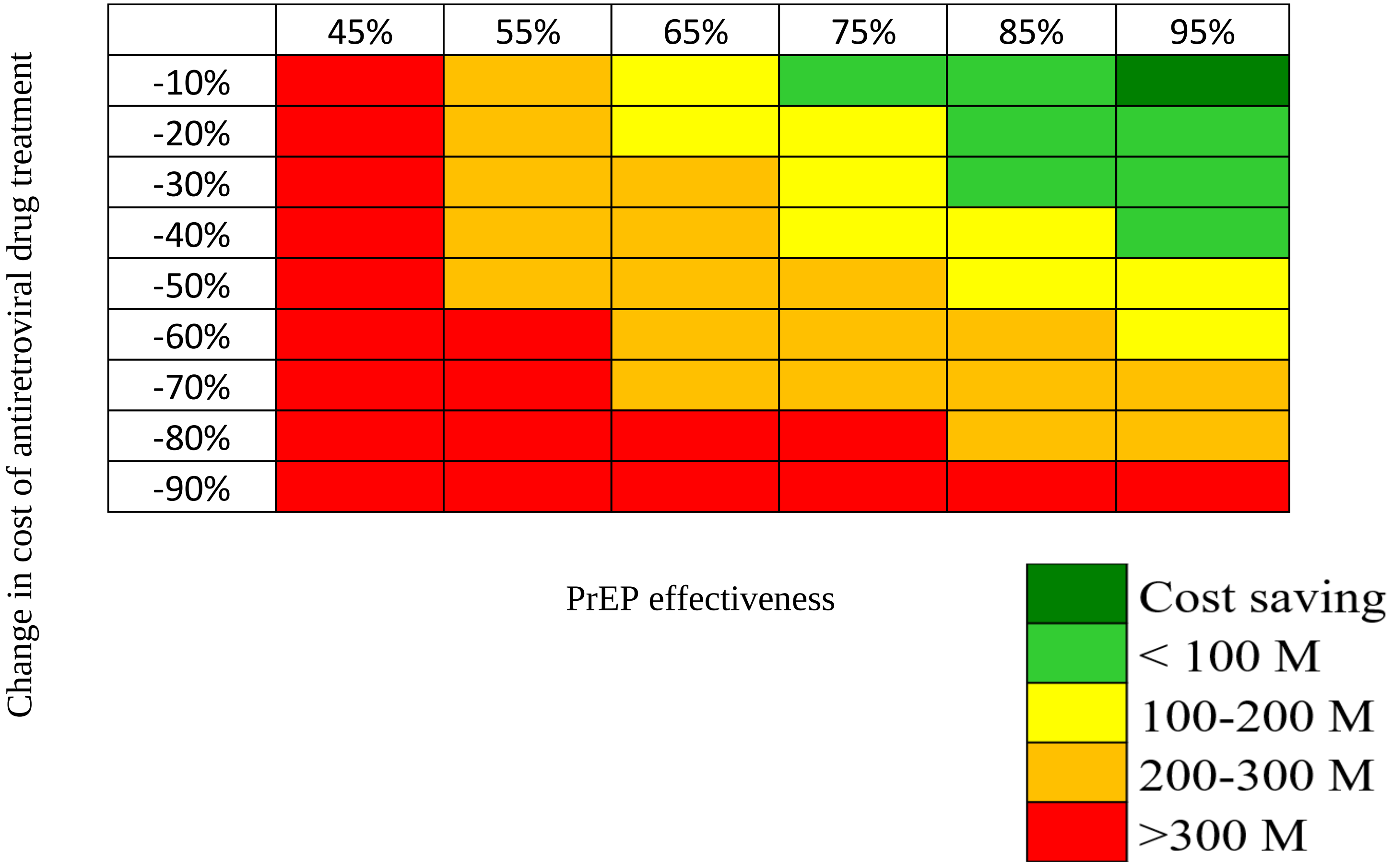

Table S7 *Minimum of years to reach break-even point in which the cumulative undiscounted costs of HIV infections averted exceed the costs of a PrEP programme. The analysis are stratified by the effectiveness of PrEP in reducing the risk of HIV infection and the future costs of antiretroviral drug treatment compared to the current costs. PrEP is assumed to be initiated in 2018. The break-even point for the discounted costs (at an annual rate of 3%) are presented in Table 3 of the main paper.*

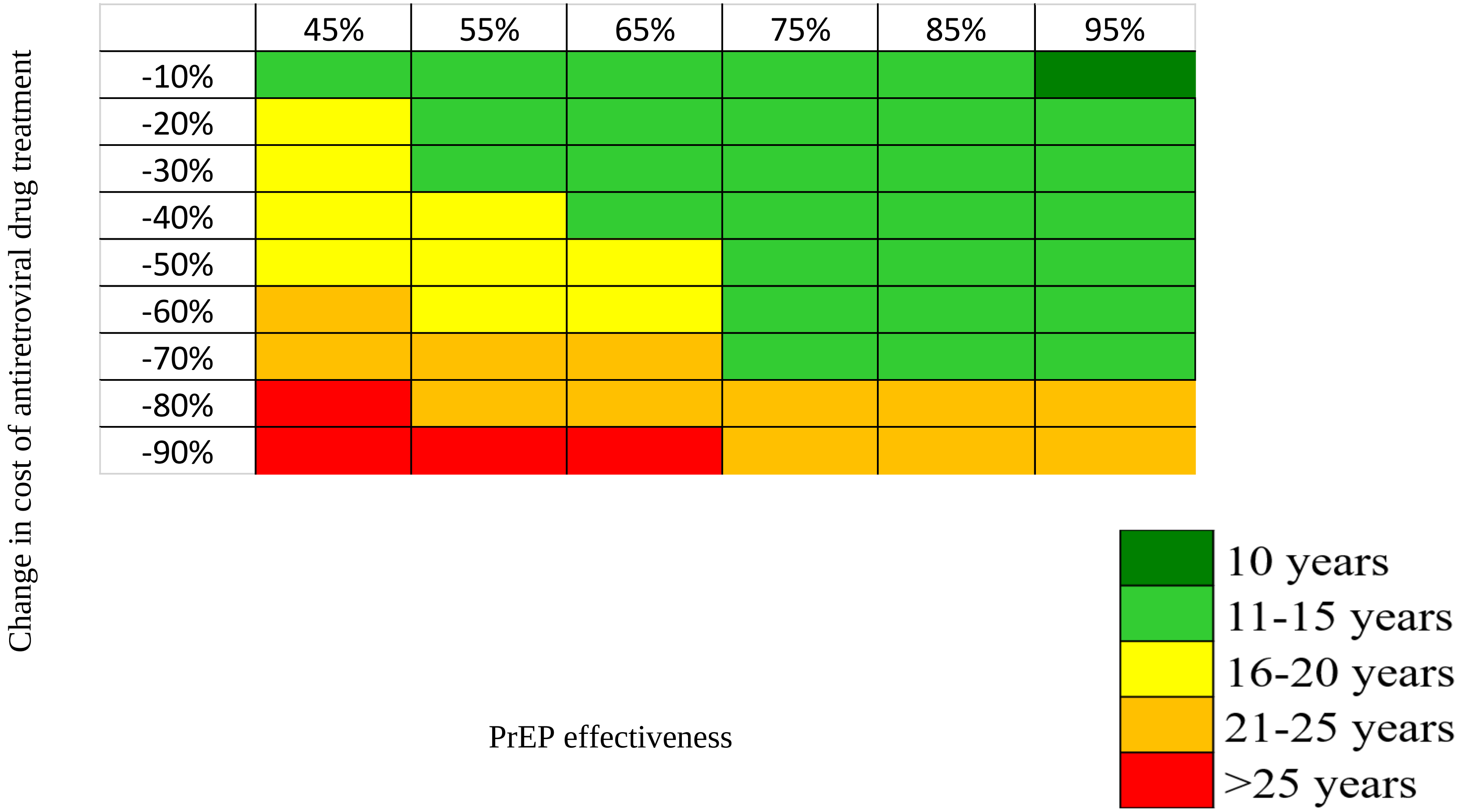

References

1. Nichols BE, Boucher CA, van der Valk M, Rijnders BJ, van de Vijver DA. Cost-effectiveness analysis of pre-exposure prophylaxis for HIV-1 prevention in the Netherlands: a mathematical modelling study. Lancet Infect Dis **2016**; 16:1423-9.
2. Nichols BE, Gotz HM, van Gorp EC, et al. Partner Notification for Reduction of HIV-1 Transmission and Related Costs among Men Who Have Sex with Men: A Mathematical Modeling Study. PLoS One **2015**; 10:e0142576.
3. Keeling MJ, Rohani P. Modeling infectious diseases in humans and animals. Princeton: Princeton University Press, **2008**.
4. Garnett GP, Anderson RM. Factors controlling the spread of HIV in heterosexual communities in developing countries: patterns of mixing between different age and sexual activity classes. Philos Trans R Soc Lond B Biol Sci **1993**; 342:137-59.
5. Marcus U, Hickson F, Weatherburn P, Schmidt AJ, Network E. Estimating the size of the MSM populations for 38 European countries by calculating the survey-surveillance discrepancies (SSD) between self-reported new HIV diagnoses from the European MSM internet survey (EMIS) and surveillance-reported HIV diagnoses among MSM in 2009. BMC Public Health **2013**; 13:919.
6. Lodi S, Phillips A, Touloumi G, et al. Time From Human Immunodeficiency Virus Seroconversion to Reaching CD4+ Cell Count Thresholds <200, <350, and <500 Cells/mm3: Assessment of Need Following Changes in Treatment Guidelines. Clin Infect Dis **2011**; 53:817-25.
7. Life expectancy of individuals on combination antiretroviral therapy in high-income countries: a collaborative analysis of 14 cohort studies. Lancet **2008**; 372:293-9.
8. Robert Koch Institut. Schätzung der Prävalenz und Inzidenz von HIV-Infektionen in Deutschland, Stand Ende 2014. Epidemiologisches Bulletin **2015**; 45:475-90.
9. Robert Koch Institut. Epidemiologisches Bulletin 38. HIV-Jahresbericht 2015. Vol. 38. Berlin: Robert Koch Institut, **2016**.
10. Robert Koch Institut. Schätzung der Zahl der HIV-Neuinfektionen und der Gesamtzahl von Menschen mit HIV in Deutschland. Stand Ende 2016. Epidemiologisches Bulletin **2016**:531-45.
11. Gesellschaft DAGDÖA. Deutsch-Österreichische Leitlinien zur antiretroviralen Therapie der HIV-Infektion **2017**.
12. National Association of Statutory Health Insurance Physicians (Kassenärztliche Bundesvereinigung KVB). Onlie Version of the Uniform Value Scale (Online Version des Einheitlichen Bewertungsmaßstab EBM). Available at: http://www.kbv.de/html/online-ebm.php. Accessed 12.03.2017 2017.
13. Mostardt S, Hanhoff N, Wasem J, et al. Cost of HIV and determinants of health care costs in HIV-positive patients in Germany: results of the DAGNA K3A Study. The European journal of health economics : HEPAC : health economics in prevention and care **2013**; 14:799-808.
14. Deutsche AIDS-Gesellschaft (DAIG). Deutsch-Österreichische Leitlinien zur antiretroviralen Therapieder HIV-Infektion. Available at: http://www.awmf.org/leitlinien/detail/ll/055-001.html. Accessed 17.10.2017.
15. LAUER-FISCHER GmbH. WEBAPO® InfoSystem. Available at: https://www.cgm.com/lauer-fischer/loesungen_lf/lauer_taxe_lf/webapo_infosystem_lf/webapo_infosystem.de.jsp. Accessed 19.07.2017.
16. Robert Koch-Institut Abteilung für Infektionsepidemiologie. HIV-Serokonverterstudie am Robert Koch-Institut. Eine Studie zur Ermittlung der Einflussgrössen auf den Verlauf der HIV-Erkrankung, der Verbreitung von HIV-Subtypen sowie der Übertragung resistenter Viren. Available at: http://www.rki.de/DE/Content/InfAZ/H/HIVAIDS/Epidemiologie/Surveillance/SeroKon/Serokonverterstudie.html. Accessed 17.10.2017.
17. Federal Office of Statistics (Statistisches Bundesamt). Harmonisierter Verbraucherpreisindex (inkl. Veränderungsraten): Deutschland, Jahre. Available at: https://www-genesis.destatis.de/genesis/online/data;jsessionid=F50037A5E2FCEDC8499216ECFCAE0D2F.tomcat_GO_2_3?operation=begriffsRecherche&suchanweisung_language=de&suchanweisung=Harmonisierter+Verbraucherpreisindex. Accessed 19.07.2017.
18. Tengs TO, Lin TH. A meta-analysis of utility estimates for HIV/AIDS. Med Decis Making **2002**; 22:475-81.
19. Venables W, Ripley B. Modern Applied Statistics with S. 4th ed.: Springer, **2002**.
